# Plasmalemmal Vesicle Associated Protein (PLVAP) as a therapeutic target for treatment of hepatocellular carcinoma

**DOI:** 10.1186/1471-2407-14-815

**Published:** 2014-11-06

**Authors:** Yun-Hsin Wang, Tsung-Yen Cheng, Ta-Yuan Chen, Kai-Ming Chang, Vincent P Chuang, Kuo-Jang Kao

**Affiliations:** Department of Research, Koo Foundation Sun Yat-Sen Cancer Center, Lih-Der Road, Taipei, Taiwan; Department of Surgery, Koo Foundation Sun Yat-Sen Cancer Center, Lih-Der Road, Taipei, Taiwan; Department of Radiology, Koo Foundation Sun Yat-Sen Cancer Center, Lih-Der Road, Taipei, Taiwan

**Keywords:** PLVAP, Hepatocellular carcinoma, Monoclonal antibody, Tissue factor, Thrombotic treatment

## Abstract

**Background:**

Hepatocellular carcinoma (HCC) is a malignancy with poor survival outcome. New treatment options for the disease are needed. In this study, we identified and evaluated tumor vascular PLVAP as a therapeutic target for treatment of HCC.

**Methods:**

Genes showing extreme differential expression between paired human HCC and adjacent non-tumorous liver tissue were investigated. PLVAP was identified as one of such genes with potential to serve as a therapeutic target for treatment of HCC. A recombinant monoclonal anti-PLVAP Fab fragment co-expressing extracellular domain of human tissue factor (TF) was developed. The potential therapeutic effect and toxicity to treat HCC were studied using a Hep3B HCC xenograft model in SCID mice.

**Results:**

PLVAP was identified as a gene specifically expressed in vascular endothelial cells of HCC but not in non-tumorous liver tissues. This finding was confirmed by RT-PCR analysis of micro-dissected cells and immunohistochemical staining of tissue sections. Infusion of recombinant monoclonal anti-PLVAP Fab-TF into the main tumor feeding artery induced tumor vascular thrombosis and extensive tumor necrosis at doses between 2.5 μg and 12 μg. Tumor growth was suppressed for 40 days after a single treatment. Systemic administration did not induce tumor necrosis. Little systemic toxicity was noted for this therapeutic agent.

**Conclusions:**

The results of this study suggest that anti-PLVAP Fab-TF may be used to treat HCC cases for which transcatheter arterial chemoembolization (TACE) is currently used and potentially avoid the drawback of high viscosity of chemoembolic emulsion for TACE to improve therapeutic outcome. Anti-PLVAP Fab-TF may become a viable therapeutic agent in patients with advanced disease and compromised liver function.

**Electronic supplementary material:**

The online version of this article (doi:10.1186/1471-2407-14-815) contains supplementary material, which is available to authorized users.

## Background

Worldwide, primary liver cancer is the fifth most common cancer in men and the seventh in women. An estimated 748,300 new liver cancer cases occurred during 2008 [1]. Approximately 695,500 people died from liver cancer that same year. Globally, HCC is the second leading cause of cancer death in men and the sixth leading cause among women. HCC accounts for 85% of primary liver cancer [2] and is endemic in Southeast Asia and Sub-Saharan Africa. Although HCC is uncommon in western countries, incidence of the disease increased two fold between 1985 and 1998 and is expected to increase until 2020 in the United States [3]. Despite its relatively low incidence, HCC is the fifth and the ninth leading cause of cancer deaths for men and women, respectively, in the US [4]. The five year overall survival rate for patients with HCC is only 15% [5].

Early stage solitary HCC can be treated with surgical resection, ablative intervention (radiofrequency ablation and ethanol injection) or liver transplantation. Intermediate stage HCC can be treated with transcatheter arterial embolization (TAE) or chemoembolization (TACE). Treatment using TACE has been shown to prolong survival [6]. However, this treatment approach has drawbacks. TACE often cannot distribute chemotherapeutic drugs (which are emulsified in lipiodol oil) evenly and thoroughly in tumors, particularly larger tumors due to high viscosity of chemoembolic emulsion [7]. The infused embolization particles (e.g. Ivalon™, Gelfoam) and chemotherapeutic agents also can damage hepatic arteries, cause blood vessel occlusion, and prevent patients from receiving further TAE or TACE treatment for recurrent disease. The newer radiolabeled microspheres are effective to control disease progeression but associated with radiation-induced injury to liver, lung and gastrointestinal tract [8–10]. HCC patients with liver cirrhosis and impaired liver function are often precluded from treatment using cytotoxic chemotherapeutic agents [11, 12]. Although targeted therapy with sorafenib is beneficial for some patients through probable disruption of tumor angiogenesis, the effectiveness of the treatment has been modest [13]. Further improvement in treatment of HCC patients with intermediate and advanced stage disease is urgently needed.

Through a comparative study of gene expression in paired tumor and adjacent non-tumorous tissues, we discovered that PLVAP protein was specifically expressed in vascular endothelial cells of HCC and not in vascular endothelial cells of non-tumorous liver tissue. This differential expression of PLVAP provides a potential target for HCC treatment. We therefore developed a recombinant monoclonal anti-PLVAP Fab fragment that co-expresses a water-soluble extracellular domain of human tissue factor (anti-PLVAP Fab-TF), which was shown effective to treat HCC in a Hep3B xenograft model using SCID mice.

## Methods

### Animal use and human subject

The use of small vertebrate animals was approved by the Koo Foundation Sun Yat-Sen Cancer Center Institutional Animal Care and Use Committee (ID number 20100908-1). The de-identified gene-expression dataset and the de-identified paraffin tissue blocks with histological diagnosis of HCC were obtained from the central institutional depository and used in the present study. The study was approved and granted exemption of informed consent by the Koo Foundation Sun Yat-Sen Cancer Center Institutional Review Board (ID number 20060731A).

### Identification of differentially expressed genes in HCC

Gene expression profiles of eighteen pairs of frozen fresh HCC and adjacent non-tumorous liver tissues were determined using the Affymetrix GeneChip Human Genome U133A array as reported [14]. The tissues used in this dataset were collected from surgically excised liver for treatment of HCC. Genes that showed extreme differential expression between paired HCC and adjacent non-tumorous liver tissues were identified by following the steps described below. Affymetrix MAS5.0 and dChip (version 2004) softwares were both used to define expression status of each gene as ‘present’ , ‘absent’ or ‘marginal’ in all 18 pairs of tissues. Tumor-specific genes showing extreme differential expression were defined as genes classified as ‘present’ by both MAS 5.0 and dChip softwares in HCC tissue and ‘absent or marginal’ in the paired adjacent non-tumorous liver in at least 16 out of 18 pairs of these tissues. By adopting such an approach, we identified two tumor-specific genes that showed extreme differential expression between HCC and adjacent non-tumorous liver tissue. One was PLVAP and the other was MELK. The microarray dataset has been deposited at the Gene Expression Omnibus under accession number GSE60502.

### Laser capture microdissection

Laser capture micro-dissection (LCM) of formalin fixed HCC tissue sections was carried out using the Arcturus PixCell^R^ IIe system, CapSure™ HS LCM caps, and the Paradise™ reagent system from Arcturus Bioscience, Inc. (Mountain View, CA). First, seven micrometer thick tissue sections were de-paraffinized, rehydrated, and stained for LCM according to the manufacturer’s instructions. Target cells were captured on CapSure™ HS LCM caps using a 7.5-μm spot-size laser set at 50 mW power and 1.3 ms duration. Approximately 5000 to 6000 HCC cells without vascular endothelium or adjacent non-tumorous liver were captured on each cap and prepared for RNA extraction. Additionally, vascular endothelial cells were carefully dissected from the HCC tissue. Only 1000 to 2000 HCC vascular endothelial cells were captured for RNA extraction due to their relative paucity.

### RNA extraction and real time quantitative RT-PCR for PLVAP mRNA

Cells captured on LCM caps were used for RNA extraction, cDNA synthesis, *in vitro* transcription and antisense RNA amplification using the Paradise™ reagent system in accordance with the manufacturer’s instructions. The first reverse transcription step was carried out using 4.5 μl anti-sense RNA and TaqMan Reverse Transcription Reagents (Applied Biosystems, Carlsbad, California) in a final volume of 10 μl according to the manufacturer’s protocol. The second step of real-time PCR was performed using 2.4 μl of cDNA template, TaqMan primers/probe mix and universal PCR Master Mix (Applied Biosystems) in a final volume of 25 μl. Real-time PCR was performed using a Smart Cycler II (Cephid, Inc., Sunnyvale, CA). Reactions were initially incubated at 50°C for 2 minutes and then at 95°C for 10 minutes. Thereafter, there were 45 cycles of denaturation at 95°C for 15 seconds and annealing/extension at 60°C for 40 seconds. The primer and probe sequences are listed in (Additional file 1: Table S1).

### Immunohistochemical staining for PLVAP expression

A murine anti-human PLVAP monoclonal antibody (GY5 mAb), which was developed in-house, was used to study PLVAP expression in HCC and non-tumorous liver tissue. This mAb binds to a linear antigenic epitope corresponding to amino acids 331 to 441 of human PLVAP protein. To study murine PLVAP expression using Hep3B tumor xenografts, rat anti-mouse PLVAP mAb prepared from MECA32 hybridoma supernatant was used [15]. This hybridoma was obtained from the Developmental Studies Hybridoma Bank at the University of Iowa (Iowa City, IA). Immunohistochemical staining was performed using a Benchmark XT automated stainer (Ventana Medical Systems, Inc., Tucson, AZ). After antigen retrieval and blocking of endogenous peroxidase, tissue sections were incubated with 1 μg/ml anti-human or 5 μg/ml anti-mouse PLVAP monoclonal antibody at 37°C for 48 minutes. The sections were then processed using the *i*View™ DAB Detection Kit (Ventana Medical Systems). When performing IHC using rat anti-mouse mAb, biotinylated rabbit anti-rat IgG (AbD Serotec, Oxford, UK) was used to replace the biotinylated second antibodies in the *i*View™ DAB Detection Kit.

### Establishing Hep3B xenografts in SCID mice

**Figure 1:**
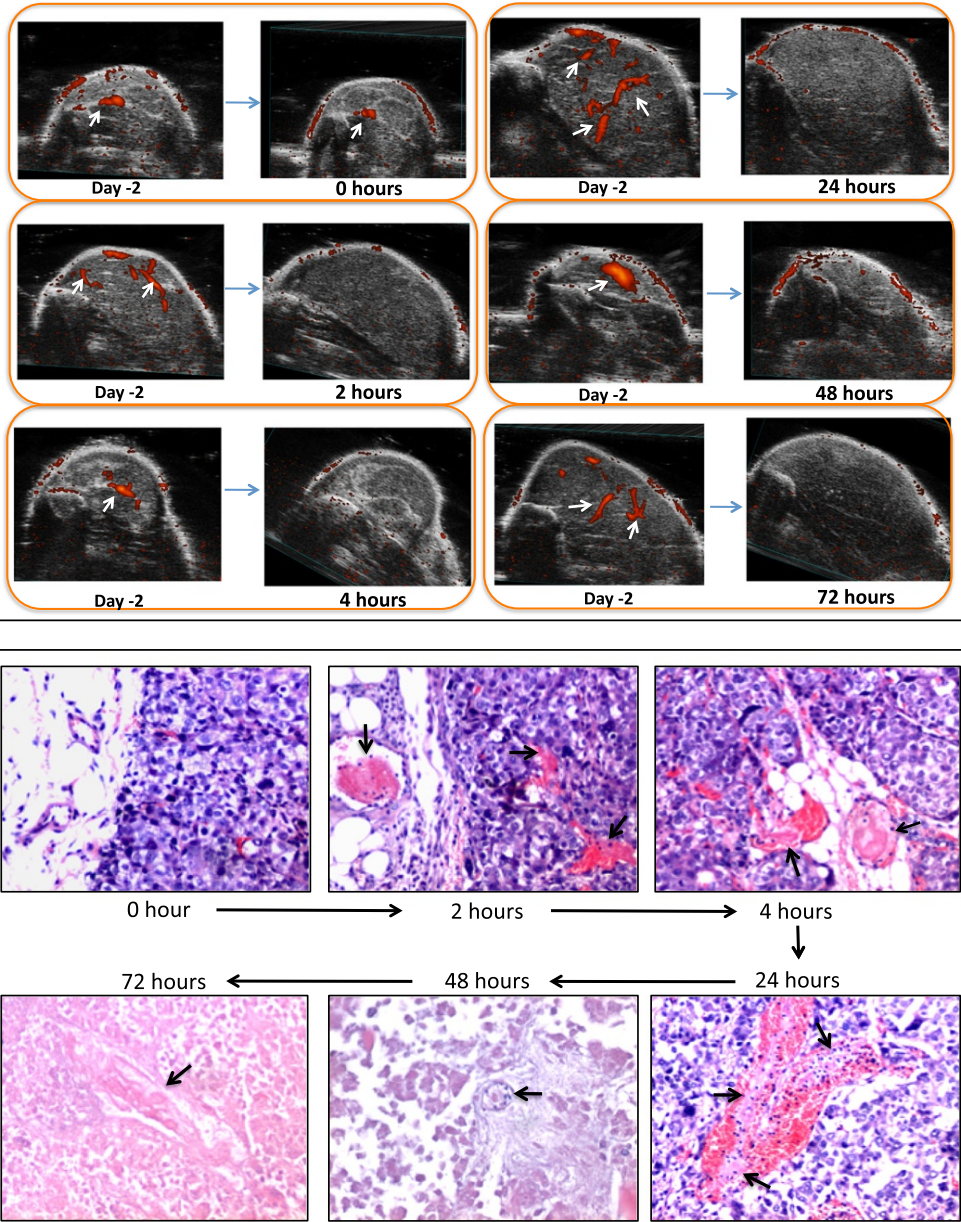
**Changes of tumor blood flow and tumor histology at 2, 4, 24, 48 and 72 hours after treatment with 10 μg MECA32-Fab-TF.** Tumor blood flow was monitored using 3D power Doppler sonography (upper panel). The histology sections were stained with hematoxylin and eosin (lower panel). There were two mice at each time point. Results were the same between two mice at each time point. Only result from one of the two mice studied at each time point is shown. Upper panel shows change of tumor blood flow before and after treatment. White arrows point at blood flow signal in tumors. Blood flow signal disappeared at 2 hours and persisted up to 72 hours. Lower panel shows that fibrin thrombi (balck arrows) in blood vessels became evident at 2 hours after treatment and persisted throughout the study period. Tumor tissue became morphologically degenerated at 24 hours. Frank necrosis became evident at 48 hours. Photomicrographs were taken at 100x magnification.

**Figure 2:**
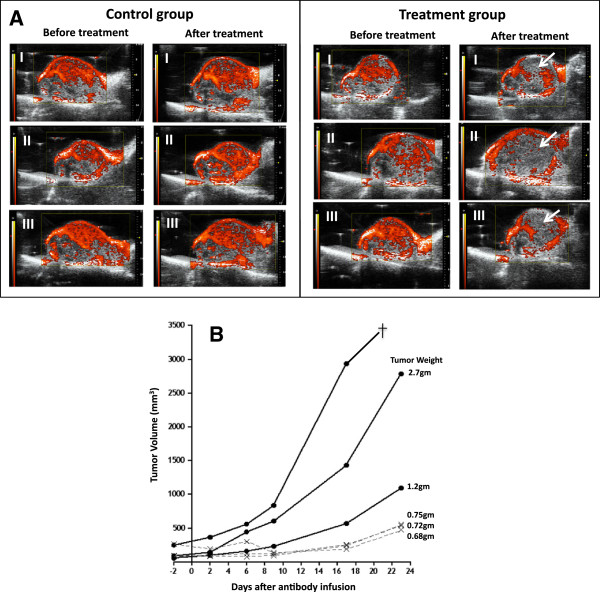
**Blood supply and tumor growth in Hep3B tumor xenografts after intra-arterial infusion of 20 μg MECA32 mAb chemically conjugated with human tissue factor (MECA32-TF) into a tumor feeding femoral artery.** Control mice were infused with 20 μg MECA32 mAB. **A**: Power Doppler was performed 48 hours before and after the treatment. Red signals in tumors represent blood flow, which were significantly diminished in mice after treating with MECA32-TF (white arrow) but not in those treated with control MECA32 mAb. **B**: Tumor growth before and after treatment. Solid circles (•) are control mice and crosses (x) are mice treated with MECA32-TF. †: Death.

To establish a HCC xenograft model in BALB/c C.B-17 SCID mice, 4 million Hep3B cells were subcutaneously injected at the right inner thigh of SCID mice. Hep3B cells were cultured in DMEM media containing 10% fetal bovine serum, 1% GlutaMax™, 1x antibiotic-antimycotic and 10 mM HEPES. All cell culture reagents were purchased from Life Technologies (Grand Island, NY). Cells were treated with EDTA solution (Life Technologies) and harvested upon reaching 80% confluence. After being washed with serum-free DMEM, Hep3B cells were suspended in ice cold serum-free DMEM containing 75% Matrigel (BD Biosciences, San Jose, CA) at a concentration of 66.7 million cells per milliliter. After subcutaneous injection of four million Hep3B cells suspended in Matrigel, it took the injected tumor cells 5 to 6 weeks to grow and become ready for study. Initially, tumor sizes were manually monitored each week using an electronic caliper. Later, a Vevo 2100 3D Ultrasound Imaging System (Visual Sonics, Toronto, Canada) was used. Blood flow in Hep3B tumors was assessed by 3D power Doppler using the same ultrasound imaging system. To compare blood flow before and after treatment, the same parameters were used for sonography and power Doppler before and after treatment in the same experiment. To reduce background noise further, the sensitivity setting used for power Doppler experiment of Figure 1 was lower than that used in the experiment of Figure 2.

### Chemical conjugation of recombinant GST-hTF to MECA32 rat anti-mouse PLVAP mAb

Purified MECA32 mAb was dialyzed in 0.1 M 2-(N-morpholino) ethanesulfonic acid (MES) buffer containing 0.5 M NaCl at pH 6.0. The antibody was adjusted to 1 mg/ml. Additionally, 1 ml of MECA32 mAb, 1.2 mg EDC (1-Ethyl-3-[3-dimethylaminopropyl]carbodiimide hydrochloride) and 3.3 mg of sulfo-NHS (N-hydroxysulfosuccinimide) were added. After gentle vortexing to dissolve the added reagents, the mixture was incubated at room temperature for one hour. A Zeba desalting column (Thermo Fisher Scientific Inc., Rockford, IL) pre-equilibrated with PBS coupling buffer was used to recover activated MECA32 mAb. Next, an equal mole of GST-hTF (0.33 mg in 0.66 ml) was added to the activated MECA32-mAb. The mixture was incubated on a rotary mixer for 3 hours at room temperature. The reaction was then quenched by adding hydroxylamine to a final concentration of 10 mM. The antibody conjugated with human tissue factor protein was extensively dialyzed against 1x phosphate buffered saline. The concentration of antibody was determined by absorbance at 280 nm using an extinction coefficient of 1.37 for 1 mg/ml. The antibody conjugated with human tissue factor (MECA32-TF) was characterized for its tissue factor activity using a chromogenic substrate assay [16], and for binding to mouse PLVAP using an ELISA assay (Additional file 2: Supplementary Methods). The production of water soluble and truncated forms of GST-hTF and mouse PLVAP proteins is detailed in the Supplementary Methods.

### Production of a recombinant anti-mouse PLVAP Fab fragment co-expressing hTF

To produce a therapeutic biologic with a well-defined structure and stoichiometry between anti-PLVAP mAb and hTF, a recombinant anti-murine PLVAP Fab fragment co-expressing hTF at the carboxyl terminus of the Fd chain was developed. The Fab fragment of this therapeutic biologic was derived from MECA32 mAb. The procedures for preparation of MECA32 anti-PLVAP Fab-TF recombinant protein (MECA32-Fab-TF) are detailed in the Additional file 2. The purified MECA32-Fab-TF was analyzed using SDS-PAGE and characterized for PLVAP binding activity and human tissue specific activity before use.

### Arterial infusion for treatment in Hep3B tumor xenografts

To demonstrate the therapeutic effect of MECA32-TF and MECA32-Fab-TF on Hep3B xenografts, different doses of MECA32-TF or MECA32-Fab-TF were infused into the main tumor feeding femoral artery. Mice carrying Hep3B tumor xenografts were anesthetized by isoflurane (Baxter, Guayana, Puerto Rico) inhalation and laid in the supine position under a dissecting microscope. The hair over the right inguinal area was removed with Nair™ hair remover (Church & Dwight, Inc. Ewing, NJ) 24 to 48 hours before infusion. After cleansing the skin with 75% alcohol, a 0.5-cm incision was made at the right inguinal area just above the tumor. The right femoral artery and vein were exposed, and the femoral artery was then looped with a 6-0 nylon thread. The artery was gently retracted proximally. An arteriotomy was performed using a micro-scissor distal to the retraction and a fine 33-gauge needle was inserted into the vascular lumen. MECA32-TF, MECA32-Fab-TF or control MECA32 antibody was infused at a rate of approximately 40 μl per minute. Injection was performed under a dissecting microscope to ensure that there was no leakage. After infusion, the needle was withdrawn and the arteriotomy site was sealed with Histoacryl (TissueSeal, Ann Arbor, MI). The nylon for retraction was removed. After confirmation of adequate hemostasis, the incision was closed using a continuous suture.

### Staistical methods

All statistical analyses were carried out using the R software package (v2.6) from Bioconductor (http://www.bioconductor.org). Descriptive statistics, analysis of variance, and linear mixed-effect model analysis [17] were used to analyze results obtained from different experiments as indicated.

## Results

### Identification of PLVAP as a therapeutic target for HCC

**Figure 3:**
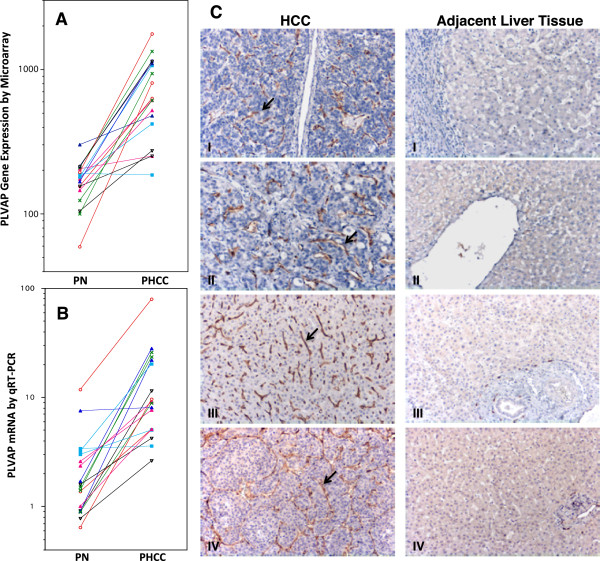
**Differential expression of PLVAP between paired HCC tissue and adjacent non-tumorous liver tissue. A**: Differential expression of the PLVAP gene according to microarrays of 18 pairs of HCC and adjacent non-tumorous liver tissue. PN: paired non-tumorous liver; PHCC: paired HCC tissue. **B**: Relative quantities of PLVAP mRNA in the same 18 tissue pairs. One non-tumorous liver tissue sample was chosen as a reference control (relative quantitative expression = 1). **C**: Immunohistochemical (IHC) staining of PLVAP in four randomly selected HCC cases. IHC staining was performed using GY5 murine anti-human PLVAP monoclonal antibody. Endothelial cells lining blood vessels of HCC showed positive staining for PLVAP in brown color (arrows). IHC staining (panel **C**) showed that PLVAP was not expressed by the endothelial cells of hepatic central vein (C-II right panel), hepatic sinusoid (CI-IV right panels), and hepatic arterioles (portal tract) (C-III right panel) in the adjacent non-tumorouse liver tissues. The large empty space in the right panel of C-II was lumen of a hepatic central vein which showed absence of PLVAP expression in the lining endothelial cells. We also stained HCC sections including adjacent non-tumorous liver with anti-human CD34 monoclonal antibody. Endothelial cells of hepatic central vein and hepatic areteriole were stained positively for CD34 expression (data not shown). Liver sinusoidal endothelial cells did not express CD34 as expected.

To identify genes specifically expressed in HCC and not in non-tumorous liver tissue, we compared the gene expression profiles of 18 pairs of HCC and adjacent non-tumorous liver tissues. HCC-specific gene expression was defined as expression of a gene determined to be “present” in HCC and “absent or marginal” in adjacent non-tumorous liver tissues by both MAS5.0 and dChip softwares in at least 16 of the 18 pairs of tissue samples. Using this stringent approach only two genes met the criteria. One was PVLAP and the other was MELK. After further examining PLVAP expression in all 18 tissue pairs (Figure 3A), we found that all pairs but one showed higher PLVAP expression in the HCC tissues. The differential expression of PLVAP in HCC was confirmed in 16 out of the same 18 tissue pairs using TaqMan real time quantitative RT-PCR (Figure 3B).

PLVAP is a known structural protein of the endothelial stomatal and fenestral diaphragms and is found in specialized vascular endothelial cells [18, 19]. To confirm that PLVAP expression in HCC tissue was indeed confined to vascular endothelial cells, laser capture micro-dissection was used to harvest tumor vascular endothelial cells and tumor cells from two different cases of formalin-fixed paraffin-embedded HCC tissue blocks (Additional file 1: Figure S1). We also dissected adjacent non-tumorous liver cells and sinusoid-lining endothelial cells (Additional file 1: Figure S1). RNA extracted from the dissected cells was analyzed for PLVAP gene expression using TaqMan real time quantitative RT-PCR. The results indicated that PLVAP gene expression in HCC tissue was restricted to tumor vascular endothelial cells (Table 1 and Additional file 1: Figure S2). HCC tumor cells and non-tumorous liver tissues did not express significant amount of PLVAP.

**Table 1:** **Quantification of PLVAP mRNA in HCC vascular endothelial cells, HCC tumor cells and adjacent non-tumorous liver tissue using laser-capture microdissection and Taqman real time quantitative RT-PCR**

	Quantity of PLVAP mRNA relative to HCC endothelial cells		
HCC Sample	HCC vascular endothelial cells	HCC tumor cells	Adjacent non-tumorous hepatocytes and sinusoid
I	100%	0.2%	0%
II	100%	0.60%	0.1%

The tracings of Taqman real time quantitative RT-PCR are shown in Additional file 1: Figure S1.

Two randomly selected HCC samples were studied.

The specific expression of PLVAP by tumor vascular endothelial cells was further confirmed by immunohistochemical staining using monoclonal anti-human PLVAP antibodies (Figure 3C). We found that PLVAP is expressed in vascular endothelial cells of HCC but not in the endothelial cells of the liver sinusoid, central vein, portal vein or hepatic arteriole (Figure 3C). PLVAP expression was not detected in vascular endothelial cells of the metastatic colon or ovarian cancer in the liver (data not shown). PLVAP was neither detected in vascular endothelial cells taken from focal nodular hyperplasia of the liver. These findings indicated that PLVAP was differentially expressed in vascular endothelial cells of HCC. Therefore, PLVAP could serve as a useful therapeutic target for treatment of HCC. Using immunohistochemical staining, we also found that endothelial cells of mouse blood vessels grown in a Hep3B xenograft expressed murine PLVAP (Additional file 1: Figure S3).

### Targeting PLVAP to treat HCC xenografts using MECA32-TF

To determine whether PLVAP expression in tumor vascular endothelial cells could be used as a target for treating Hep3B xenograft, monoclonal MECA32 anti-PLVAP antibody was chemically cross-linked with the extracellular domain of human tissue factor (MECA32-TF). Tissue factor is a potent trigger of blood coagulation protein [20, 21]. The prepared MECA32-TF had 385 μg TF activity per mg of conjugated antibody. Each SCID mouse bearing a Hep3B tumor xenograft was infused with 20 μg MECA32-TF into a tumor feeding artery. The control group was treated with 20 μg MECA32 mAb. The effect on tumor blood flow and growth before and after infusion was monitored using 3D power Doppler sonography. The results demonstrated that infusion of 20 μg MECA32-TF led to tumor blood flow blockage (Figure 2A) and suppressed tumor growth (Figure 2B). In contrast, there was no blockage of tumor blood supply or growth in the control group (Figure 2).

### Production and characterization of recombinant MECA32-Fab-TF

Although MECA32-TF prepared by chemical conjugation was therapeutically active, SDS-PAGE indicated that the number of TF protein molecules cross-linked to each MECA32 mAb was heterogeneous. To create a therapeutic anti-PLVAP antibody with a well-defined structure and a lower molecular weight to shorten circulation half-life and limit potential adverse effects, we developed a recombinant monoclonal MECA32 Fab fragment co-expressing the extracellular domain of human tissue factor protein at the carboxyl end of the Fd fragment (MECA32-Fab-TF) (Additional file 1: Figure S4). This recombinant Fab-TF had a molecular weight of 81 kDa. Based on six different batches of MECA32-Fab-TF, the average tissue factor specific activity was 90 ± 22 μg (mean ± SD) in each mg of MECA32-Fab-TF and the average binding affinity to recombinant PLVAP protein (Kd) was 5.7 ± 1.4 × 10^-8^ M (mean ± SD) using a steady state binding assay and Scatchard analysis (Additional file 2).

### Effect of anti-PLVAP MECA32-Fab-TF on Hep3B tumor xenografts within 72 hours of treatment

SCID mice bearing human Hep3B tumor xenografts at the right inner thigh were infused with 10 μg MECA32-Fab-TF into the main tumor feeding femoral artery under a dissecting microscope. The treated SCID mice were sacrificed at 0, 2, 4, 24, 48 and 72 hours after treatment. There were two mice at each time point. Power Doppler was used to monitor tumor blood flow before and after treatment. Necropsy was performed and tumors were harvested for histological examination. The results of power Doppler imaging showed tumor blood flow blockage at 2 hours after treatment, and this effect persisted throughout the 72-hour study period (Figure 1). Histological examination of the tumors indicated that thrombi with fibrin-like deposits were discernible in tumor blood vessels 2 hours after infusion. Blood vessels with thrombi present in tumor capillaries and venules became more prominent at four and twenty four hours after treatment. At 24 hours, tumor cells began to show loss of cohesiveness. At 48 hours, frank ischemic necrosis became evident. The textbook histological criteria were used to assess necrosis [22]. These findings as shown in Figure 1 suggest that infusion of anti-PLVAP MECA32-Fab-TF into the main tumor feeding artery triggered thrombosis in tumor blood vessels, blocked tumor blood flow and caused ischemic necrosis of tumors. We did not find any bleeding at the incision site in any of the treated mice. No gross adverse systemic effects were noted.

**Figure 4:**
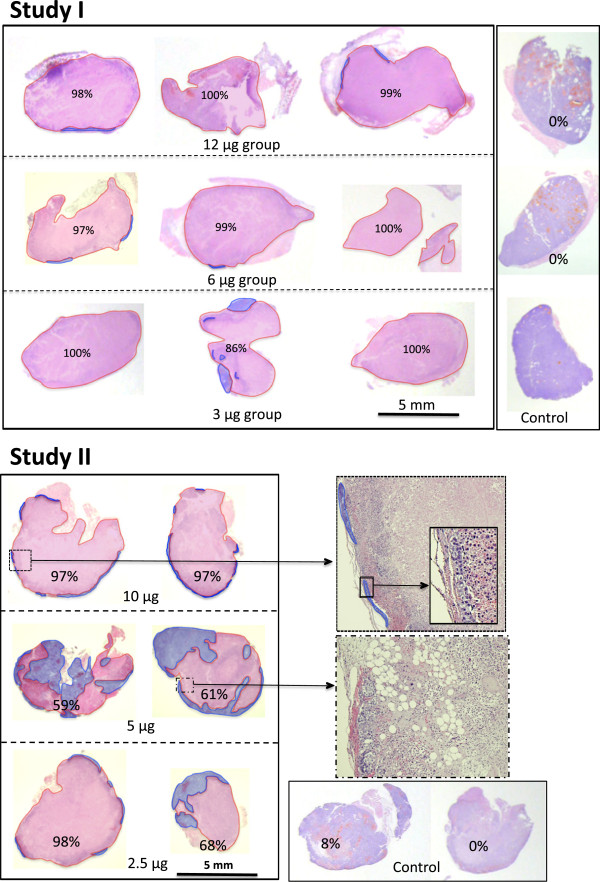
**Tumor necrosis 72 hours after infusion of different doses of MECA32-Fab-TF.** The results of two different studies are shown here. The largest tumor cross sections were submitted for histology and studied. Necrotic tumors and viable residual tumors were outlined as areas of pink and blue, respectively. The relative size of necrotic and viable tumor tissue was measured based on two dimensional areas. Percentages shown in the figure represent relative necrotic area in tumor sections. In study I, all three control tumors at right showed no necrosis (0%). In study II, photomicrographs of residual viable tumor and adjacent necrotic tumor tissue are shown at a higher magnification of 12.5x on the right. A 40x magnification to show few layers of residual viable tumor cells is shown in the inset.

Next, we studied tumor necrosis induced by different doses of MECA32-Fab-TF in two separate experiments. Tumor necrosis was assessed 72 hours after treatment. As shown in Figure 4, a dose as low as 2.5 to 3 μg was sufficient to induce 68% to nearly 100% necrosis in tumor xenografts. The results of these two studies indicated that infusion of 10 μg MECA32-Fab-TF could more consistently induce near total necrosis of tumors with an average size approximately 0.2 ml (Figure 4).

### Effect of anti-PLVAP MECA32-Fab-TF on growth in Hep3B tumor xenografts

**Figure 5:**
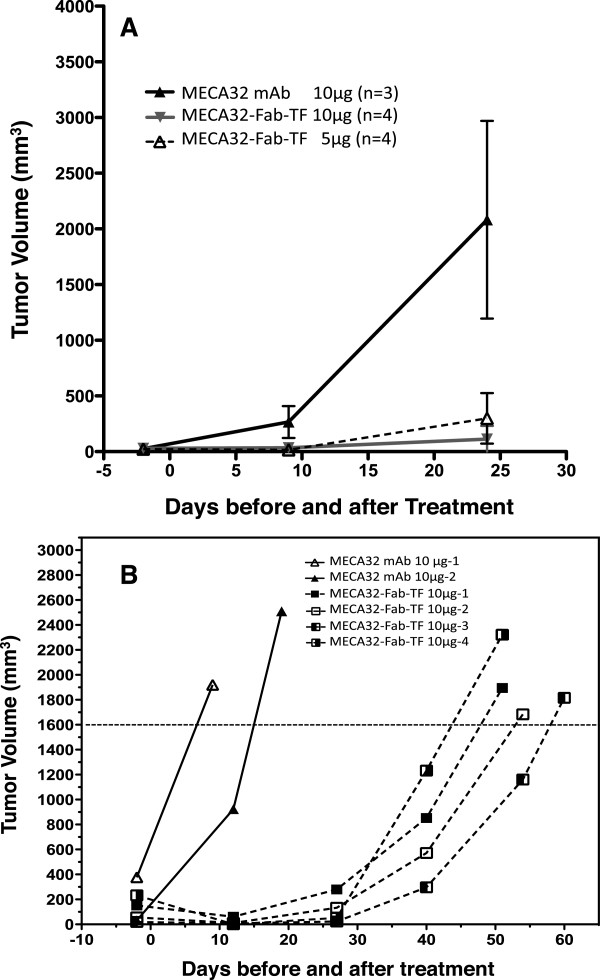
**Tumor growth after infusing MECA32-Fab-TF or control MECA32 mAb into a tumor feeding artery.** The results of two different studies were shown here. In study **A**, tumor bearing mice were treated with 5 or 10 μg MECA32-Fab-TF or 10 μg MECA32 mAb. All mice were euthanized 24 days after treatment. The growth rates between the treatment groups and the control group were compared using a linear mixed-effects model. Significant differences in tumor growth between controls and 5 μg or 10 μg treatment groups were noted (p = 0.003 and 0.001). In study **B**, tumor bearing mice were treated with 10 μg MECA32-Fab-TF (n = 4) or control MECA32 mAb (n = 2). Mice were sacrificed when tumors grew large enough to interfere with movement and food intake. The average numbers of days required to reach a tumor size of 1600 mm^3^ for control and treatment groups were 9.8 and 51.8 days, respectively. Different rates of tumor growth were noted between experiments and between mice within the same experiments. Therefore, effort was made to match tumor sizes between control and treatment groups in each study.

We then studied the effect of MECA32-Fab-TF treatment on tumor growth. Two different studies were conducted. The first study followed tumor growth for 25 days after treatment, at which point the tumors in the control group grew too large and the study was stopped. Tumor growth was monitored using 3D sonography. SCID mice bearing Hep3B xenografts were treated with 5 μg or 10 μg of MECA32-FAb-TF and controls were treated with 10 μg of MECA32 mAb without tissue factor. The results, shown in Figure 5A, demonstrate that a single dose of 5 μg or 10 μg MECA32-Fab-TF effectively suppressed tumor growth; this effect was not observed in mice given 10 μg MECA32 mAb as a control. Power Doppler study again revealed significant reduction of tumor blood flow 2 hours after treatment with MECA32-Fab-TF, but not in control mice treated with MECA32 mAb.

In the second study, SCID mice bearing Hep3B tumor xenografts were treated with intra-arterial infusion of 10 μg MECA32-Fab-TF (n = 4) or 10 μg MECA32 mAb (n = 2). When Hep3B tumors grew to approximately 2000 mm^3^, tumor-bearing mice were euthanized. This study allowed us to assess any delay of tumor growth in the treatment group. The results, summarized in Figure 5B, indicated a significant delay of tumor growth after one single infusion of 10 μg MECA32-Fab-TF into a tumor feeding artery. The average number of days after injection before tumors grew to 1600 mm^3^ were 9.8 ± 3.0 days and 51.8 ± 3.2 days for the control and treatment mice, respectively. The results of these two studies indicate that infusion of anti-PLVAP MECA32-Fab-TF into the tumor feeding artery is therapeutically effective for inducing tumor necrosis and suppressing tumor growth.

### Effect of systemic administration of anti-PLVAP MECA32-Fab-TF on growth of Hep3B tumor xenografts

To determine whether the therapeutic effect of MECA32-Fab-TF could be achieved through systemic administration, we studied the effect of intravenous injection of 10 or 20 μg of MECA32-Fab-TF through a tail vein into a SCID mouse bearing a Hep3B tumor xenograft. The control group was injected with PBS buffer. Tumor volume was monitored after treatment on day 0. The final tumor volumes among all three groups were compared by analysis of variance and there were not significantly different differences (p = 0.96). The average tumor volumes were 1844 ± 840 mm^3^ (control, n = 3), 1867 ± 602 mm^3^ (20 μg MECA32-Fab-TF, n = 3) and 1617 ± 559 mm^3^ (10 μg MECA32-Fab-TF, n = 3). Thus, infusion of MECA32-Fab-TF into a tumor feeding artery was necessary to achieve the therapeutic effect.

### Toxicity and pharmacokinetic studies of MECA32-Fab-TF

To determine the safety profile of MECA32-Fab-TF, we administered 100 μg MECA32-Fab-TF through a tail vein in each mouse. The amount injected was 10 times of an upper therapeutic dose. In this study, four male and four female 8-week-old mice were divided into four groups. Each group consisted of 1 male and 1 female. Before and after injection, mice were bled for complete blood counts and plasma MECA32-Fab-TF concentration. Coagulation factor X and fibrinogen levels were also measured to assess possible intravascular consumption of these coagulation factors. Groups I, II, III, and IV were bled at 30 seconds, 10 minutes, 30 minutes and 24 hours after injection, separately. Groups I and II were bled again on day 4. Groups III and IV were bled on day 6. After treatment, the treated mice were closely monitored for possible bleeding and body weight loss for 2 weeks. The result of our study showed a short circulation half-life of 25 minutes for MECA32-Fab-TF. There was transient reduction of plasma factor X to 30% of baseline value at 30 minutes after injection. Platelet counts also showed transient reduction at 30 minutes and were recovered at 96 hours after injection. There was no significant change of plasma fibrinogen level or body weight. These results are summarized in Additional file 1: Figure S5.

## Discussion

The use of TF to trigger thrombosis of tumor blood vessels and induce tumor necrosis was reported by Huang et al. [23]. The authors demonstrated that tumor cells engineered to secrete gamma interferon induced expression of major histocompatibility complex (MHC) class II antigens in tumor vascular endothelial cells. The induced expression of class II MHC antigens can be used as targets to treat tumor using bi-specific antibody against class II MHC antigens and water-soluble form tissue factor. The bi-specific antibody carried tissue factor to tumor blood vessels and induced thrombosis. However, this approach was applicable only to tumor cells engineered to secrete gamma interferon. The lack of naturally occurring specific targets in tumor vascular endothelial cells limits the applicability of such a therapeutic approach.

The high degree of differential expression of PLVAP we identified in HCC vascular endothelial cells offered an ideal target to test whether anti-PLVAP antibody coexpressing human tissue factor can be used to treat HCC. Due to technical infeasibility to infuse our therapeutic biologic into tumor feeding hepatic artery in mice, we established a HCC xenograft model with blood supply from femoral artery. in SCID mice. The results of our study demonstrate that recombinant anti-PLVAP Fab-TF is able to achieve therapeutic effects as anticipated. In addition to HCC, anti-PLVAP Fab-TF potentially may be used for the treatment of malignant glioma. Similar to HCC, PLVAP was highly expressed in vascular endothelial cells of glioma, but not in vascular endothelial cells of normal brain tissue [24].

In our study, the number of mice used in each experiment was limited, because infusion of anti-PLVAP Fab-TF into hair size tumor feeding femoral artery is technically challenging and has precluded us from having a larger number of mice in each experiement. Nevertheless, consistent results were obtained in many different experiments. We administered anti-PLVAP Fab-TF through a tumor feeding artery out of concern of systemic toxicity. It is known that PLVAP is expressed in many normal organs and tissues, including the endocrine glands, digestive organs, kidneys, lungs and others [25]. We reasoned that infusion of anti-PLVAP Fab-TF into the tumor feeding artery would provide a saturating concentration of anti-PLVAP Fab for binding to the target antigen and reduce the amount of Fab-TF required to achieve therapeutic effect through systemic administration. Despite the presence of PLVAP in various normal organs and tissues, we did not find any significant adverse effects after arterial infusion. Histological examination of organs did not reveal any pathology. The low systemic toxicity of anti-PLVAP Fab-TF was further supported by our systemic administration of a high dose of anti-PLVAP Fab-TF through a tail vein in mice (Additional file 1: Figure S5). The lack of systemic toxicity was likely due to the short half-life of Fab-TF, the dilution of anti-PLVAP Fab-TF in systemic circulation, and the extensive presence of PLVAP in the lungs and other organs. The amount of anti-PLVAP Fab-TF that bound to the endothelial cells of normal organs might be too low and quickly inactivated by tissue factor pathway inhibitor.

After knowing that anti-PLVAP Fab-TF had little systemic toxicity, we tested the therapeutic effects of anti-PLVAP Fab-TF via systemic administration. However, we were unable to achieve the same therapeutic effect when administering anti-PLVAP-Fab-TF through a tail vein. The lack of therapeutic effect via systemic administration may have been due to an insufficient amount of anti-PLVAP-Fab-TF reaching the tumor target for the same reasons that systemic administration did not elicit any system toxicity. Our study suggests that the therapeutic effects of anti-PLVAP-Fab-TF for treatment of HCC best be achieved through infusion into a tumor feeding artery similar to the current TACE/TAE procedures.

Our finding that arterial infusion was necessary to achieve therapeutic effect differs from an earlier study reported by Huang et al. [23]. The authors of that study showed that systemic intravenous injection of a bi-specific antibody to class II MHC antigen and human TF had induced tumor necrosis. This discrepancy was likely due to differential distributions of the targeted antigens. Expression of the class II MHC antigens used by Huang et al. was exclusively restricted to tumor vascular endothelial cells, and class II MHC antigens were not present in the endothelial cells of normal organs or tissues. Therefore, no class II MHC antigens in normal organs or tissues to compete for binding of bi-specific antibody to tumor vascular endothelial cells. The absence of competition allowed the injected bi-specific antibody to be gradually accumulated in tumor tissue. In our case, PLVAP was present in many other non-hepatic organs, which may prevent effective accumulation of TF in tumors when anti-PLVAP Fab-TF was administered systemically. Therefore, anti-PLVAP Fab-TF was not therapeutically effective through systemic administration and required direct administration into tumor feeding artery to achieve its therapeutic effect.

Histological examination of the treated tumors revealed very small numbers of viable tumor cells remaining at the tumor edge (Figure 4). This finding is not unexpected because tumor cells at the edges of HCC can receive collateral blood supply from surrounding tissue making them resistant to thrombotic blockage of tumor blood vessels. To further prevent re-growth of the residual tumor cells at the tumor edges, anti-angiogenic therapy after anti-PLVAP Fab-TF treatment is an attractive option and warrants further study.

Current major approaches for treating intermediate stage HCC rely mainly on transcatheter arterial injection of chemoembolic agents or radiolabeled embolic spheres, local ablation using radiofrequency heating and/or intra-tumoral alcohol injection. The effectiveness of these approaches is often limited by size, number, shape and anatomical location of targeted tumors. There are also inherent limitations to each of these therapeutic modalities. For instance, chemoembolic agents and radiolabeled embolic spheres are not HCC specific and can produce bystander cytotoxicity [8–10]. It is also difficult to control the distribution of viscous emulsion and embolic particles within tumors during theses procedures. The shunting of therapeutic agents from tumor blood vessels into normal liver and systemic circulation can lead to unwanted complications. In cases of advanced stage HCC, patients often have to rely on systemic chemotherapy or targeted therapy (e.g. sorafenib). Unfortunately, severely compromised liver function in these patients often precludes them from receiving cytotoxic chemotherapy. Targeted therapy using sorafenib provides only a modest survival benefit to some patients [13]. The limitations and challenges of existing systemic treatments and the development of new targeted systemic treatment have been recently reviewed [26, 27].

We believe limitations of different therapeutic modalities mentioned above may be addressed by the use of anti-PLVAP Fab-TF. Anti-PLVAP Fab-TF possesses good fluid characteristics, high selectivity for HCC, and low systemic toxicity. The low viscosity of anti-PLVAP Fab-TF without any embolic particles may provide more even distribution and potentially more complete blockage of tumor blood flow. The low systemic toxicity of anti-PLVAP Fab-TF may also allow us to treat patients with compromised liver function and more advanced HCC.

## Conclusions

The results of our study show that there is a high degree of differential expression of PLVAP between HCC and adjacent non-tumorous liver tissue. This differential expression can be exploited to treat HCC with arterial infusion of anti-PLVAP Fab-TF using procedures similar to TACE as demonstrated in the proof of concept small animal studies reported here. The potential advantages of using anti-PLVAP Fab-TF to treat HCC include low systemic toxicity and low viscosity. These advantages may allow more HCC patients with advanced diseases eligible for treatment. Further development of anti-human PLVAP Fab-TF for trial in HCC patients is warranted.

## Electronic supplementary material

Additional file 1: Table S1: Primer and probe sequences for PLVAP and beta-actin real-time quantitative RT-PCR. **Figure S1.** Laser capture dissection of HCC vascular endothelial cells, HCC tumor cells and non-tumorous liver tissue. **Figure S2.** Real time Taqman quantitative RT-PCR tracings for PLVAP mRNA in HCC vascular endothelial cells, HCC tumor cells and adjacent non-tumorous liver tissue. **Figure S3.** Immunohistochemical staining of HEP3B tumor xenograft from SCID mouse for PLVAP expression using MECA32 anti-mouse PLVAP monoclonal antibody. **Figure S4.** Bicistronic construct for production of MECA32-Fab-TF and SDS-PAGE of purified MECA32-Fab-TF. **Figures S5-A to S5-E.** Effect of high dose of MECA32-Fab-TF (100 μg) on BALB/c mice. (PDF 7 MB)

Additional file 2: **Supplementary Methods.** (PDF 403 KB)

## Abbreviations

Fab: Fab fragment of antibody
HCC: hepatocellular carcinoma
LCM: Laser captured microdissection
mAb: Monoclonal antibody
MHC: Major histocompatibility complex
PLVAP: Plasmalemmal vesicle associated protein
TACE: Transcatheter arterial chemoembolization
TAE: Transcatheter arterial embolization
TF: Human tissue factor.
