# Supplementary material for: Plasmalemmal Vesicle Associated Protein (PLVAP) as a therapeutic target for treatment of hepatocellular carcinoma
Source: BMC Cancer. 2014 Nov 6;14:815. doi: 10.1186/1471-2407-14-815 (PMC4233082; doi:10.1186/1471-2407-14-815)

Plasmalemmal Vesicle Associated Protein (PLVAP) as a Therapeutic Target for Treatment of Hepatocellular Carcinoma

Y-H Wang, T-Y Cheng, T-Y Chen, K-M Chang, vincent P. Chuang and K-J Kao

Additional file 1
Supplementary Table and Figures

Table S1. Primer and probe sequences for PLVAP and beta-actin real-time quantitative RT-PCR.

Figure S1. Laser capture micro-dissection of HCC vascular endothelial cells, HCC tumor cells and non-tumorous liver tissue.

Figure S2. Real time Taqman quantitative RT-PCR tracings for PLVAP and beta actin mRNAs in HCC vascular endothelial cells, HCC tumor cells and adjacent non-tumorous liver tissue.

Figure S3. Immunohistochemical staining of HEP3B tumor xenograft from SCID mouse for PLVAP expression using MECA32 anti-mouse PLVAP monoclonal antibody.

Figure S4. Bicistronic construct for production of MECA32-Fab-TF and SDS-PAGE of purified MECA32-Fab-TF.

Figures S5-A to S5-E. Effect of high dose of MECA32-Fab-TF (100 μg) on BALB/c mice.

Table S1. Primer and probe sequences for real-time quantitative RT-PCR for PLVAP and beta-actin.

Gene	Forward Primer Sequence	Reverse Primer Sequence	Taqman Probe sequence
PLVAP	5'-CCTTGAGCGTGAGTGTTTCCA-3'	5'-GGCAGGGCTGGGAGTTG-3'	5'-AAGGAGTGGCTCCCCTCC-3'
beta-Actin	5'-GTCCCCCAACTTGAGATGTATGAAG-3'	5'-GTCTCAAGTCAGTGTACAGGTAAGC-3'	5'-CTCCCAGGGAGACCAA-3'

The realtive quantities of PLVAP mRNA were determiend according to “Guide to Performing Relative Quantitation of Gene Expression Using Real-Time Quantitative PCR” published by Applied Biosystems.” The guide is available at http://www3.appliedbiosystems.com/cms/groups/mcb_support/documents/generaldocuments/cms_042380.pdf.

Figure S1. Laser capture micro-dissection of HCC vascular endothelial cells, HCC tumor cells and non-tumorous liver tissue.

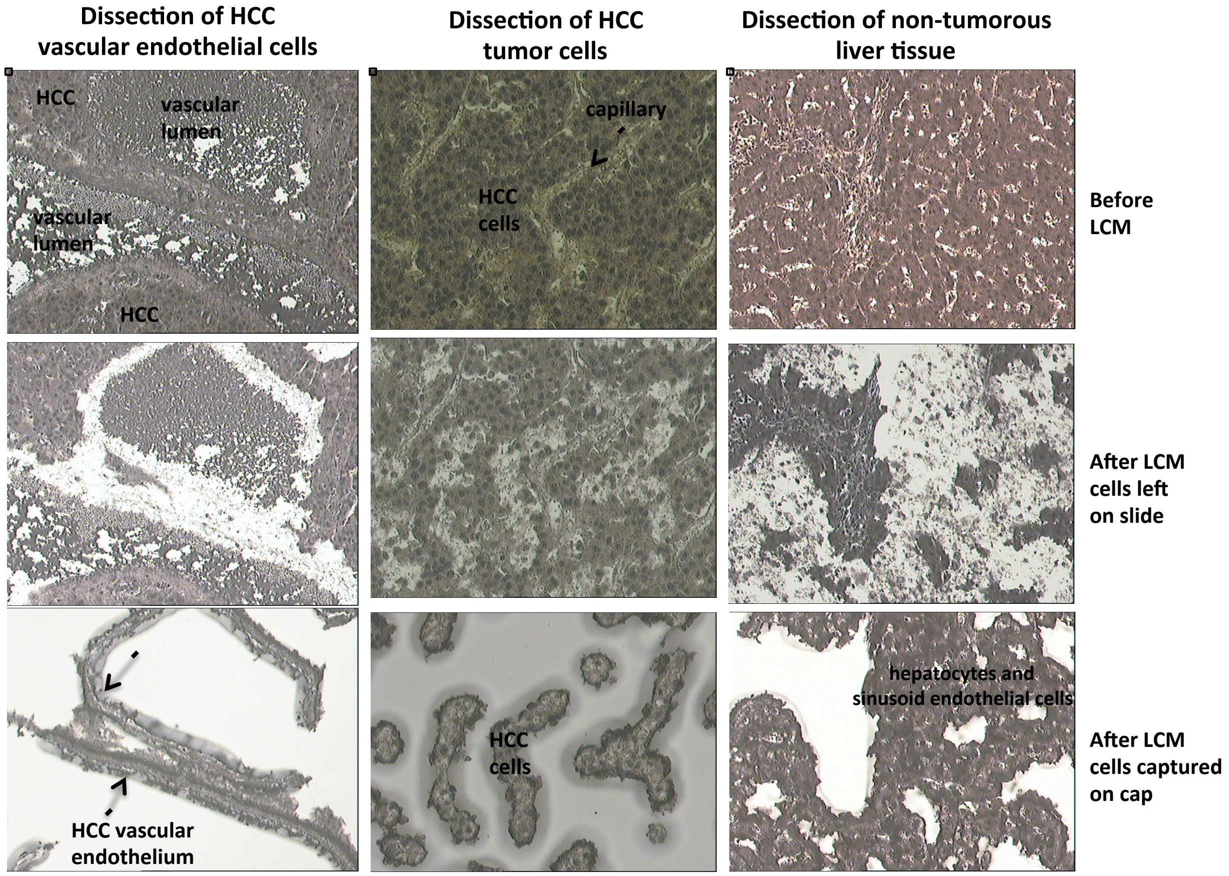

Figure S2. Real time Taqman quantitative RT-PCR tracings for PLVAP and beta actin mRNAs in HCC vascular endothelial cells, HCC tumor cells and adjacent non-tumorous liver tissue.

Two different HCC samples were studied (red and blue). Dashed lines are RT-PCR for beta actin and solid lines are RT-PCR for PLVAP.

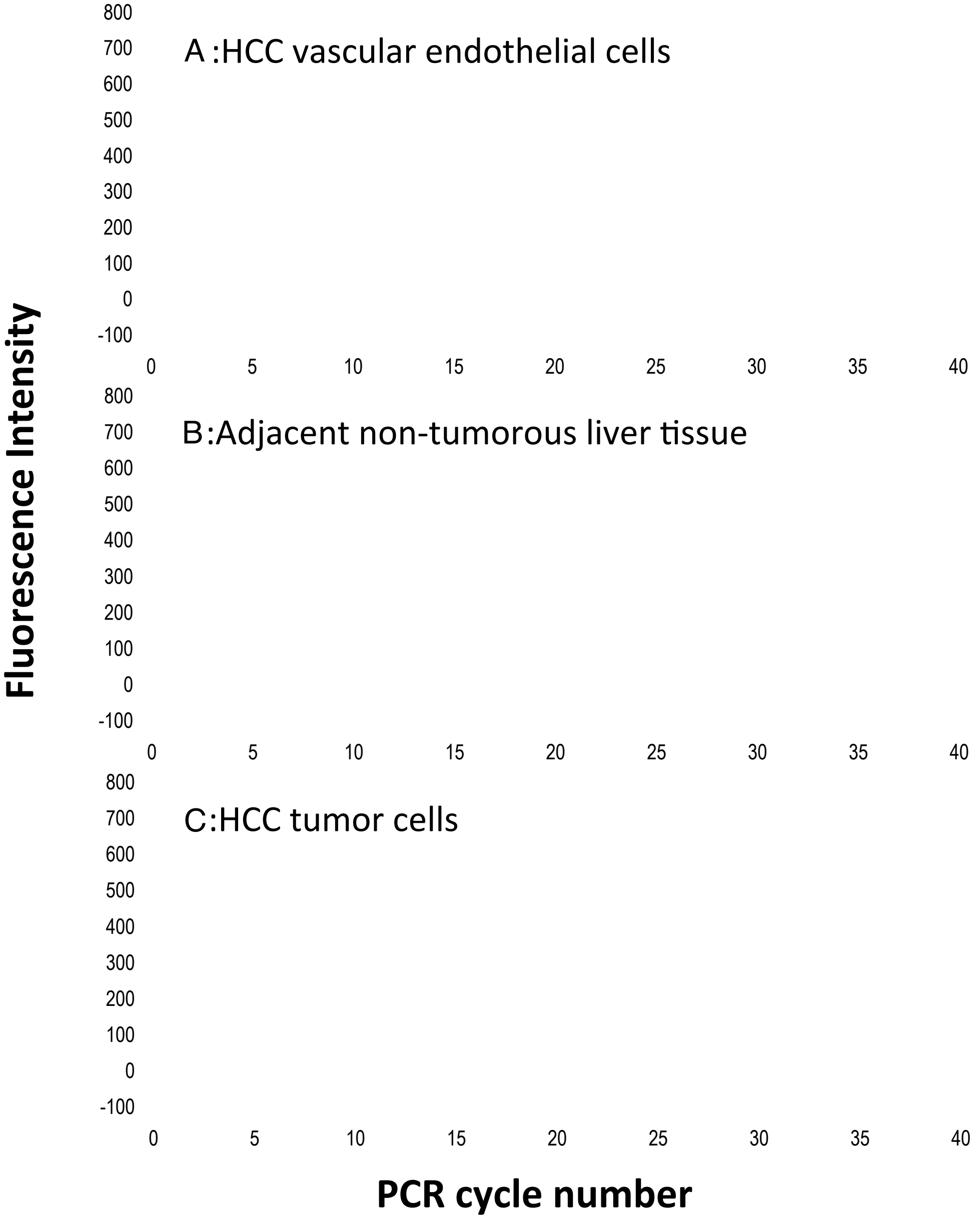

Figure S3. Immunohistochemical staining of HEP3B tumor xenograft from SCID mouse for PLVAP expression using MECA32 anti-mouse PLVAP monoclonal antibody.
Vascular endothelial cells of tumor blood vessels were stained positively in brown (arrows). The photomicrograph was taken at 100x magnification.

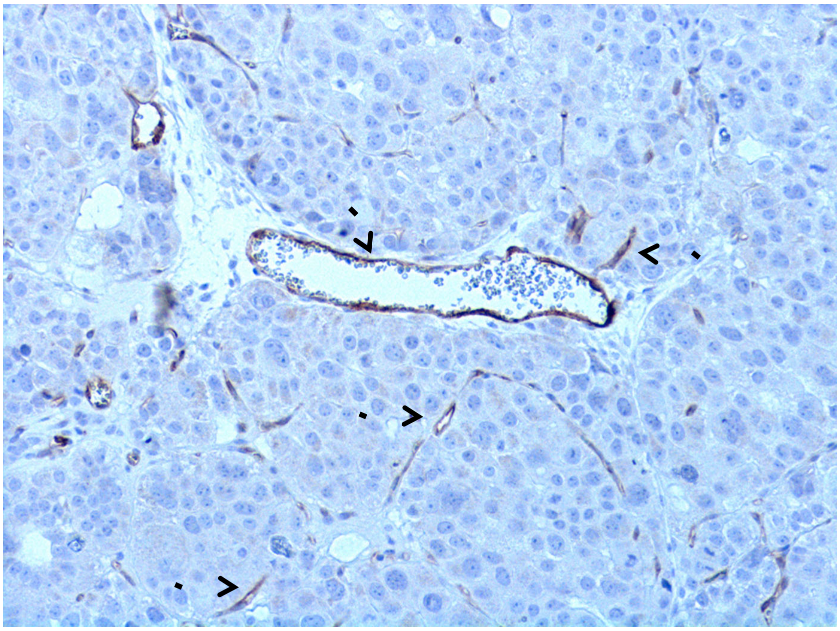

Figure S4. Bicistronic construct for production of MECA32-Fab-TF and SDS-PAGE of purified MECA32-Fab-TF.

A: The construct inserted in pET26b plasmid. RBS: ribosome binding site; VH: variable domain of IgG heavy chain; CH1: constant domain 1 of heavy chain; VK: variable domain of kappa light chain; CK: constant domain of kappa light chain; sTF: extracellular domain of tissue factor; His: histidine-tag.

B: SDS-PAGE of purified MECA332-Fab-TF and a diagram of MECA32-Fab-TF. NR: non-reducing; R: reducing.

A. Bicistronic construct for production of MECA32-Fab-TF

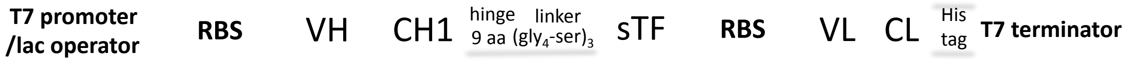

B. SDS-PAGE and protein domains of purified MECA32-Fab-TF

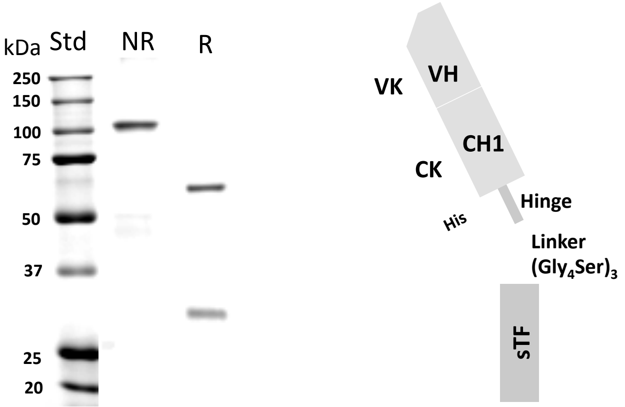

Figures S5-A to S5-E. Effect of high dose of MECA32-Fab-TF (100 µg) on BALB/c mice.

Four male and four female mice of 8 weeks old were divided into four groups. Each group consisted of 1 male and 1 female. Each mouse was injected with 100µg MECA32-Fab-TF through a tail vein. Mice before and after injection were bled for plasma concentrations of MECA32-Fab-TF (Fig. S5-A), factor X (Fig. S5-B) and fibrinogen (Fig. S5-C), and platelet count (Fig. S5-D). Groups I, II, III, and IV were bled at 30 seconds, 10 minutes, 30 minutes and 24 hours after injection, separately. Groups I and II were bled again on day 4 after injection. Groups III and IV were bled on day 6. Body weight (Fig. S5-E) and gross appearance were monitored. Mouse factor X and fibrinogen concentrations were measured using ELISA kits from Oxford Biomedical Research and Abcam, respectively. Plasma MECA32-Fab-TF was measured using an antigen-capturing ELISA.

Figure S5-A Circulation half life of MECA32-Fab-TF

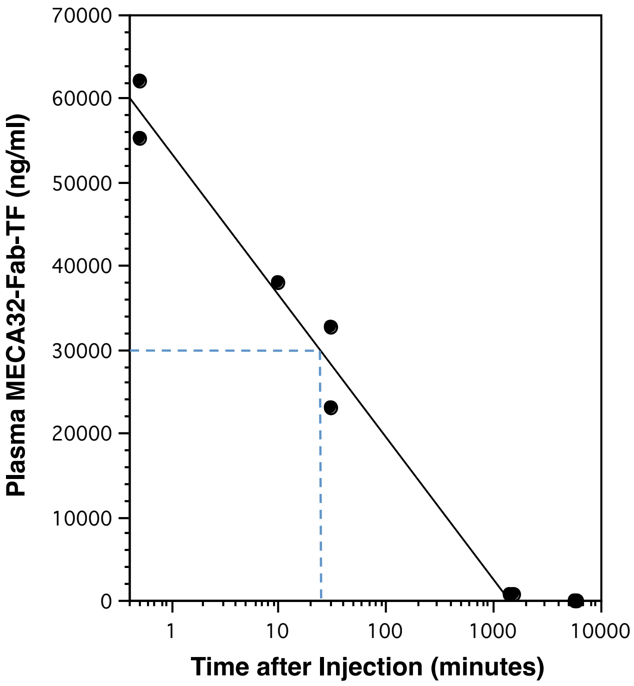

Figure S5-B Change of plasma factor X concentration after injection of 100 μg MECA32-FAB-TF. Only blood samples collected up to 96 hours after injection were studied.

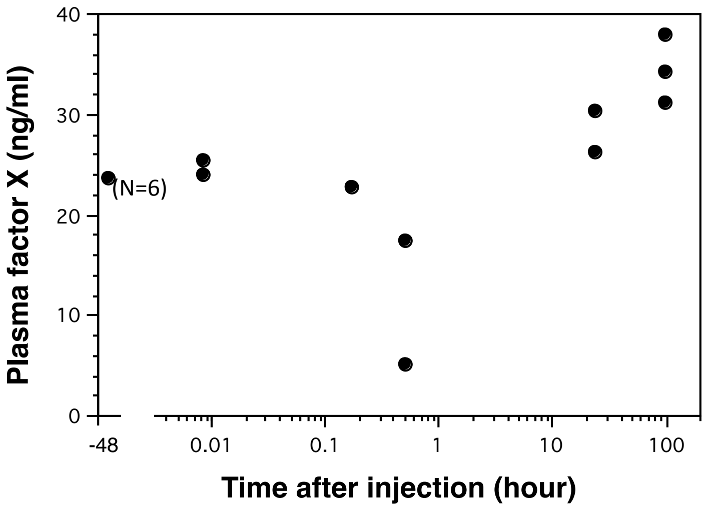

Figure S5-C Change of plasma fibrinogen concentration after injection of 100 μg MECA32-FAB-TF. Only blood samples collected up to 96 hours after injection were studied.

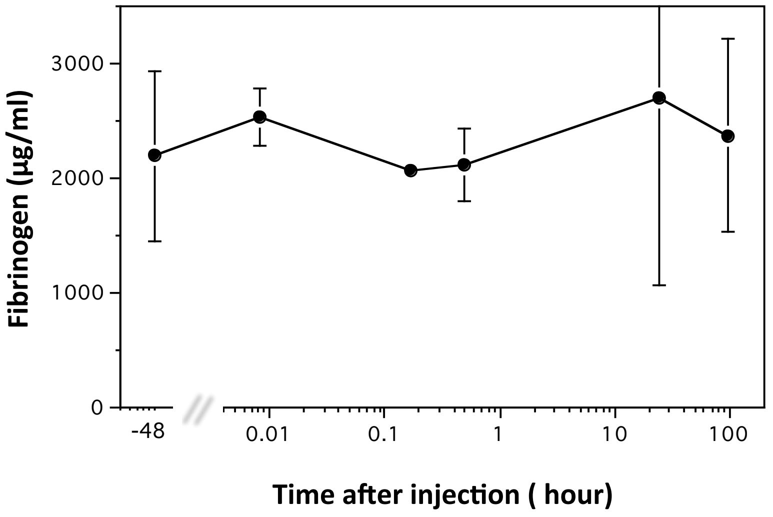

Figure S5-D Change of platelet concentration in peripheral blood after injection of 100 μg MECA32-FAB-TF

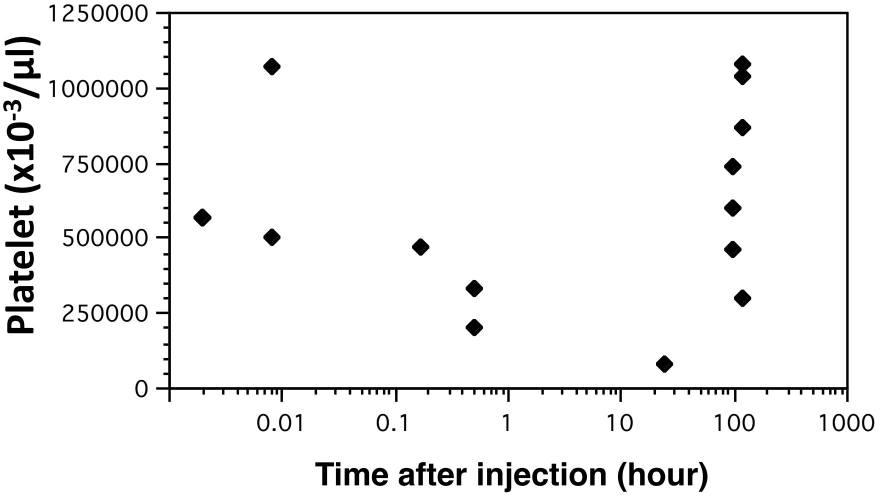

Figure S5-E Change of Body weight after injection of 100 μg MECA32-FAB-TF

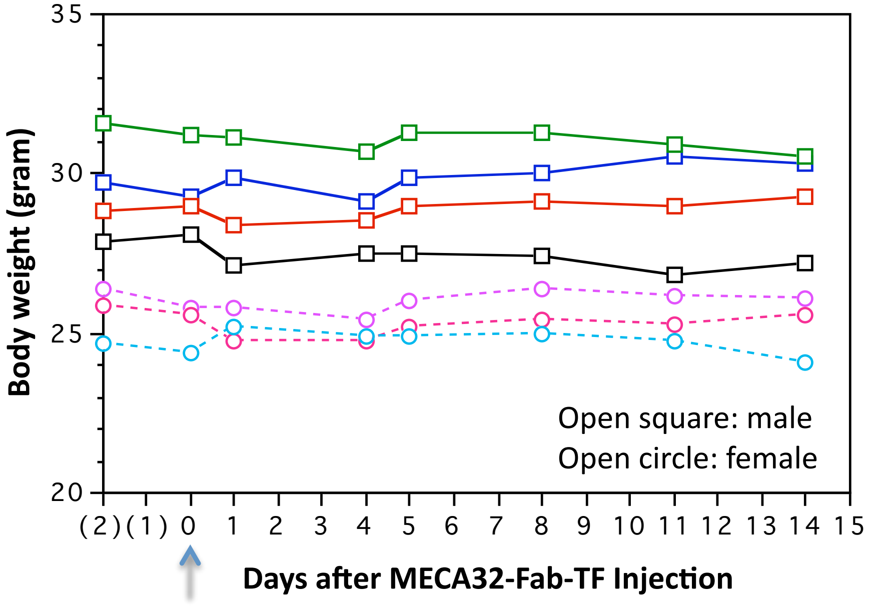

Supplement: Supplementary file 1 — Additional file 1: Table S1: Primer and probe sequences for PLVAP and beta-actin real-time quantitative RT-PCR. Figure S1. Laser capture dissection of HCC vascular endothelial cells, HCC tumor cells and non-tumorous liver tissue. Figure S2. Real time Taqman quantitative RT-PCR tracings for PLVAP mRNA in HCC vascular endothelial cells, HCC tumor cells and adjacent non-tumorous liver tissue. Figure S3. Immunohistochemical staining of HEP3B tumor xenograft from SCID mouse for PLVAP expression using MECA32 anti-mouse PLVAP monoclonal antibody. Figure S4. Bicistronic construct for production of MECA32-Fab-TF and SDS-PAGE of purified MECA32-Fab-TF. Figures S5-A to S5-E. Effect of high dose of MECA32-Fab-TF (100 μg) on BALB/c mice. (PDF 7 MB) [file 12885_2014_4997_MOESM1_ESM.pdf]
