# Supplementary material for: Plasmalemmal Vesicle Associated Protein (PLVAP) as a therapeutic target for treatment of hepatocellular carcinoma
Source: BMC Cancer. 2014 Nov 6;14:815. doi: 10.1186/1471-2407-14-815 (PMC4233082; doi:10.1186/1471-2407-14-815)
Supplement: Supplementary file 2 — Additional file 2: Supplementary Methods. (PDF 403 KB) [file 12885_2014_4997_MOESM2_ESM.pdf]

Plasmalemmal Vesicle Associated Protein (PLVAP) as a Therapeutic Target for Treatment of Hepatocellular Carcinoma

Y-H Wang, T-Y Cheng, T-Y Chen, K-M Chang and K-J Kao

Supplementary Methods

1. ELISA assay for evaluation of MECA32 anti-PLVAP monoclonal antibody (mAb) or MECA32 anti-PLVAP Fab-TF binding to recombinant murine PLVAP.
2. Production of recombinant extracellular domain of hTF protein.
3. Production of recombinant water soluble mouse PLVAP protein (mPLVAP).
4. Production of recombinant MECA32 anti-mouse PLVAP Fab fragment co-expressing hTF (MECA32-Fab-TF).
5. Human tissue factor activity assay.
6. ELISA assay for measurement of plasma MECA32-Fab-TF concentration.
7. Measurement of binding affinity for MECA32-Fab-TF

1. ELISA assay for evaluation of MECA32 anti-PLVAP monoclonal antibody (mAb) or MECA32 anti-PLVAP Fab-TF binding to recombinant murine PLVAP.

In order to make sure that the recombinant anti-PLVAP Fab-TF can bind to mouse PLVAP, an ELISA assay was used. First, each well of an ELISA plate was coated with 50 μl of 2.5μg/ml mouse recombinant PLVAP protein in PBS-azide (0.02%) overnight at 4°C. Thereafter, the assays were carried out at room temperature. After three washes of each well with 150 μl washing buffer (PBS containing 0.2% Tween-20). Each well was blocked with 150 μl blocking buffer (PBS containing 2% BSA and 0.05% Tween-20) for 30 minutes. After three washes, 50 μl of anti-mouse PLVAP MECA32 mAb or MECA32 anti-PLVAP Fab-TF was added into each well at different concentrations in duplicates. All wells were incubated for 45 minutes and washed three times. Wash well was then incubated with 50 μl biotinylated anti-human TF antibody (R&D Systems Corp.) at 1:500 dilutions in the blocking buffer for 45 minutes. After three washes, each well was incubated with 5000x diluted Streptavidin-alkaline phosphatase conjugate for 30 minutes. Each well was then incubated with 100 μl alkaline phosphatase substrate for 60 minutes and absorbance of each well was measured at 405 nm in a microplate reader.

2. Production of recombinant extracellular domain of hTF protein.

To produce recombinant water soluble hTF protein, a PCR fragment for the extracellular domain of human tissue factor cDNA (corresponding to amino acid residues 33 to 251) was prepared from a full length cDNA clone of human tissue factor (NM001993.2) (OriGene Corp., Rockville, MD). Primers used for PCR contained restriction sequences for BamHI and SalI at the 5' end of both forward and backward primers, respectively.

The amplified cDNA fragment was inserted into pGEX®-6P–1 plasmid (GE Healthcare Life Sciences) and tagged with glutathione transferase (GST). The expression construct described above was verified by DNA sequencing and transformed into *Escherichia coli* strain SHuffle™ T7 Express (New England Biolabs, Inc. Ipswich, MA) for production of hTF.

The E. Coli transformants were plated on selective medium. Later, a colony of 1-2mm was selected randomly and inoculated into 4 ml of 2xYT medium containing 100 μg/ml ampicillin at 30°C and incubate in a 230 rpm incubator shaker overnight. The following day, the overnight culture was inoculated into 400 ml of 2xYT medium containing 100 μg/ml ampicillin and continued to grow at 30°C in a 230 rpm incubator shaker overnight. When the absorbance at 600 nm reached about 0.6~0.8, Isopropyl β-D-1-thiogalactopyranoside (IPTG) was added to a final concentration of 0.4 mM to induce protein production. Shaking was continued at 30 °C for about 20 hours. Following the induction with IPTG, the cells were harvested by centrifugation (10,000 x g; 20 min) and subjected to lysis in 1x PBS with 0.2% Tween 80 containing lysozyme and Benzonase Nuclease (Novagen) at room temperature for 2 hours. Cell lysate was centrifuged at 10,000 rpm for 30 minutes at 4 °C. Supernatant was collected and filtered as soluble fraction.

The recombinant human tissue factor tagged with GST (GST-hTF) was purified from GSTrap FF column (GE Helathcare Life Sciences, Piscataway, NJ) according to the instruction of the manufacturer. The eluted fractions containing the GST-hTF were identified with SDS-polyacrylamide gel electrophoresis (SDS-PAGE), pooled and dialyzed into PBS. The concentration of the purified protein was determined using

Bradford protein assay (Bio-Rad laboratories., Hercules, CA) and bovine serum albumin as standard. The purified GST-hTF showed a protein band with expected molecular weight of 50 kDa in SDS-PAGE gel (10% polyacrylamid) as shown below. The tissue factor activity of the purified protein was assayed against a commercial human tissue factor using a chromogenic substrate assay.

SDS-PAGE of purified GST-hTF

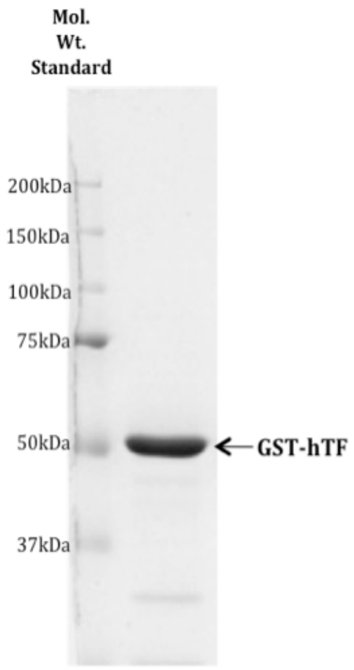

3. Production of recombinant water soluble mouse PLVAP protein (mPLVAP).

Plasmid pGEM-T Easy–mPLVAP$_{48-438}$ was generated by inserting a PCR fragment representing the truncated PLVAP (amino acid residues 48 to 438 comprising the extracellular domain of mouse PLVAP) into the pGEM®-T Easy Vector (Promega Corp.). This PCR fragment was prepared from a cDNA clone of mouse PLVAP (Invitrogen, Life Technologies Corp.) by PCR using the following primer pair:

mPLVAP CDS NdeI F : 5’CATATGTATGGCAATGTGCACGCCACC3’ and

mPLVAP Stop Xho I R:5’CTCGAGATCCACAGGTGGGCGATTCTGGC3’.

Next, a cDNA fragment representing the amino acid residues 48 to 438 of PLVAP containing NdeI and XhoI recognition sequences at each end was excised from pGEM®-T Easy –mPLVAP$_{48-438}$ and inserted into pET-15b (Novagen-EMD Millipore, Darmstadt, Germany) for protein expression. After verification by DNA sequncing, this expression construct was transformed into *Escherichia coli* (Rosetta-gami2(DE3)pLysS). Expression of His-tagged fusion mPLVAP protein in *Escherichia coli* Rosetta-gami2(DE3)pLysS was induced with 1 mM isopropyl-ß-D-thiogalactopyranoside for 16 hours at 30°C. Following the induction, the bacterial cells were subjected to lysis by sonication in equilibration buffer (50 mM sodium phosphate, 300 mM NaCl, pH 7) supplemented with 8 M urea and separated into soluble and insoluble fractions by centrifugation at 15,652 x g for 30 minutes at 4°C. To purify the His-PLVAP$_{48-438}$ protein, soluble fraction was loaded onto a TALON® Metal Affinity Resin (Clontech, Palo Alto, CA) and was eluted with elution buffer (50 mM sodium phosphate, 300 mM NaCl, pH 7, 500 mM imidazole). The resulting mouse PLVAP$_{48-438}$ protein in the eluate was dialyzed against PBS. SDS-PAGE analysis of the purified His-mPLVAP is shown below.

SDS-12% PAGE analysis of the purified His-mPLVAP

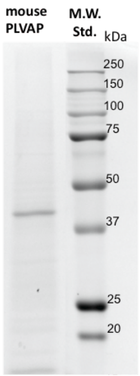

4. Production of recombinant MECA32 anti-mouse PLVAP Fab fragment co-expressing hTF (MECA32-Fab-TF).

Preparation of a plasmid construct to produce MECA32-Fab-TF was accomplished in four steps. The first step is to prepare cDNAs of variable domain of MECA32 mAb light chain (VL) and variable domain of MECA32 mAb heavy chain (VH), and determine their DNA sequences for preparation of primers to be used in the second step. The second step was to prepare full length cDNA for kappa light chain of MECA32 mAb with a His-tag at the carboxyl terminus, and inserted into pET26b plasmid vector. The third step was to prepare a cDNA of VH1 and CH1 domains (Fd) plus hinge region of MECA32 mAb heavy chain with a linker sequence at the 3' end, and cDNA for hTF and a linker sequence at the 5' end. The overlapping PCR was then used to stitch two cDNAs together. This cDNA of MECA32-Fd-hinge-linker-TF was inserted into pET26b plasmid vector. The fourth step was to construct a bicistronic plasmid vector from the plasmids prepared from the second and the third steps.

First Step. Cloning cDNAs of VL domain of MECA32 mAb kappa light chain and VH domain of MECA32 mAb heavy chain for nucleic acid sequencing. The cDNAs coding variable domains of MECA32 mAb light chain (VL) and heavy chain (VH) were prepared using FirstChoice RLM-RACE kit (Ambion, Inc., Austin, TX) according to manufacturer's instruction. Briefly, total RNA isolated from MECA32 hybridoma was used as template to amplify variable domain of light (VL) and heavy chains (VH) by reverse transcription PCR using primers complementary to the nucleotide sequences corresponding to constant domain of the kappa light chain next to VL domain (5' TGTCCTGATCAGTAACACTGTCC3') and CH1 domain of the heavy chain next to VH

doamin (5’TGAGAGTGTAGAGTCCAGACTGCAGG3’), separately.

PCR products were analyzed and isolated from the 1.5 % agarose gel using the Qiaquick gel extraction kit (Qiagen, Mississauga, Ontario, Canada). The purified PCR fragments were inserted into the plasmid vector, pGEM-T-easy (Promega, Madison, WI, USA) and transformed into Escherichia coli strain YE707-J (Yeastern Biotech, Taipei, Taiwan). Plasmids containing inserts of the VL and the VH domains were prepared from the transformed E. Coli and used for determination of DNA sequences of the VL and VH domains. The sequences then were used to design primers to be used in the second and the third steps.

Second Step. Preparation of a cDNA consisting of MECA32 mAb kappa light chain and His-tag, and inserting it into pET-26b plasmid vector. The sequence of the VL chain form the first step was used to design appropriate primer for obtaining full length kappa light chain cDNA sequence of MECA32 antibody. First, full-length kappa chain cDNA of MECA32 mAb was generated by RT-PCR from total RNA of MECA32 hybridoma cells using primers listed bellow:

Forward primer: 5’GATCCTGACATCCAGATGACCCAGACTCC3’ and

Reverse primer: 5’CACACTCATTCCTGTTGAAGCTCTTG3’.

The purified PCR fragment with BamHI and Sal I restriction sites was inserted into the plasmid vector pET26b with a $(His)_6$-tag at the carboxyl terminus of the CK domain and this plasmid was designated as pET26b-M32K.

Third Step. Preparation of MECA32-Fd-hinge-linker-TF cDNA and inserting into pET26b plasmid vector. We first prepared a cDNA consisting of MECA32 mAb FD,

hinge region plus and linker sequence by PCR using cDNA template from MECA32 hybridoma cells and the following primer pair:

Forward primer: 5'GACATCCAGATGACCCAGACTCC3' and

Hinge linker Reverse primer:

5'AGAGCCACCTCCGCCTGAACCGCCTCCACCTGTACATCCACAAGGATTGCA TTCC3'.

Next, we prepare a cDNA consisting of (Gly4Ser)3 linker sequence and extracellular domain of human tissue factor (AA. 33-251) (hTF) by PCR using cloned hTF cDNA template and the following primer pair:

hTF linker forward primer:

5'GGCGGAGGTGGCTCTGGCGGTGGCGGATCGTCAGGCACTACAAATACT GTGG3' and

TF reverse primer: 5'CAGTGTGAGGTGCAACTGGTGGAG3'.

Two PCR products were stitched by overlapping extension. The final fused PCR product was inserted into pET-26b vector. This vector was designated as pET26b-M32-Fd-TF.

Fourth Step. Construction of a biscistronic plasmid vector containing both MECA32 Fd-hinge-$(Gly_4Ser)_3$ linker-TF and MECA32 kappa light chain with a His-tag. We generated a DNA fragment by PCR using pET26b-M32-Fd-TF as template and the following primer pair.

26b-RBS-F:

5' ACAATTCCCCTCTAGATTTTGTTTAACTTTAAGAAGGAGA 3' and

26b-Termination-R:

5' CAAAATTATTTCTAGATTTCGGGCTTTGTTAGCAGCCGG 3'

This DNA fragment consisting of ribosome binding sequence (rbs), VH1 and CH1 of MECA32 mAb heavy chain, hinge region, linker sequence, hTF and stop codon. This fragment was then inserted into Xba I restriction site of pET26b-M32K. The sequence of the entire insert was verified by DNA sequencing using the dye-deoxy method. This plasmid construct was designated as pET26b MECA32-Fab-TF and transformed into the *E. coli* SHuffle T7 Express strain (New England Biolabs Corp.) for protein expression.

To produce MECA32-Fab-TF, a colony (1-2 mm) of the transformed E. Coli culture was inoculated into 4 ml of 2xYT medium containing 30μg/ml kanamycin at 30°C, 230 rpm overnight. Next morning, the overnight culture was inoculated into 400 ml of 2xYT medium containing 30μg/ml kanamycin and continued to grow at 30°C, 250 rpm. When the absorbance at 600nm reached 0.6~0.8, isopropyl β-D-1-thiogalactopyranoside (IPTG) was added to a final concentration of 0.4 mM for induction of recombinant protein production. Shaking was continued at 30°C for about 20 h.

The cells were harvested by centrifugation at 10000 x g for 20 min at room temperature and used to isolate inclusion bodies. The cell paste was suspended in 4 ml of 10 mM Tris/HCl, pH 7.5, containing 150 mM NaCl, 1 mM MgCl2, 0.17 mg/ml PMSF and 2 mg/ml hen's-egg white lysozyme (Sigma). Benzonase (250 units; EM Science) was added and the suspension was mixed gently at room temperature for 1.5 hour then centrifuged at 12000 x g for 15 min. The pellet was resuspended in 10 mM Tris/HCl, pH 7.5, containing 1 mM EDTA and 3 % Nonidet P40 (2 ml), sonicated for 1 min at 50 % power and centrifuged at 12000 g for 20 min. The pellet was re-suspended in water, sonicated for 20-30 seconds at 50 % power and centrifuged at 12000 x g for 20 min. The wash with

water was repeated, and the final pellet, highly enriched for the inclusion bodies, was suspended in buffer containing 6 M guanidinium chloride, 0.5 M NaCl, 20 mM phosphate and 10 mM 2-mercaptoethanol, pH 8 by gentle mixing at room temperature overnight. The solution was held at room temperature overnight then diluted to a protein concentration of about 1 mg/ml in 6 M urea/50 mM Tris/HC1, pH 8 and dialyzed at 4°C overnight against 10-20 volumes of the same buffer. Then, the dialysis was changed to a buffer containing 2 M urea, 50 mM Tris/HCl, 300 mM NaCl, 2.5 mM GSH, 0.5 mM GSSG, pH 8 (folding buffer). After dialysis for 2 days, the buffer was replaced with fresh folding buffer and the dialysis was continued for 2 more days. Next, dialysis buffer was changed to a buffer of 1M urea, 50mM Tris-HCl, 300 mM NaCl pH8 and the dialysis was continued for one more day. The dialysis was then carried out in the same buffer with sequentially reduced concentrations of urea from 0.8M urea for 6 hours, 0.56 M urea overnight, and 0.28 M urea for 6 hours. Finally, the dialysis was carried out in folding buffer without urea and continue overnight. The refolded supernatant was loaded onto a nickel nitrilotriacetic acid (Ni-NTA; GE Healthcare) column and eluted with 500 mM immidazol in 50 mM sodium phosphate and 0.3 M NaCl at pH7.0. Recombinant MECA32-Fab-TF was further purified by HiLoad 16/60 Superdex 75 prep grade (GE Healthcare) gel filtration column chromatography. Eluates containing target MECA32-Fab-TF were analyzed by SDS-PAGE and pooled. MECA32-Fab-TF was characterized by ELISA to confirm binding to mouse PLVAP. Tissue factor specific activity of MECA32-Fab-TF was measured using a chromogenic TF assay.

5. Human tissue factor activity assay.

Human tissue factor activity was measured using a chromogenic substrate assay. This assay is based on the ability of TF/FVIIa complex to activate factor X (FX). The amidolytic activity of the activated factor X is then measured using a chromogenic substrate. The TF activity is quantified indirectly by the amount of FXa produced. The FXa produced is measured kinetically according to the release of para-nitroamiline (pNA) from a FXa specific chromogenic substrate as change of absorbance at 405 nm.

Briefly, anti-PLVAP Fab-TF protein samples to be assayed were diluted to protein concentrations between 25 ng/ml and 150 ng/ml. Twenty microliter of recombinant TF standard (1 to 30 ng/ml) (R&D, Catalog #: 2339-PA) or diluted unknown samples was incubated with 40 ul of master mix. The master mix consisted of 10 nM human factor VIIa (HTI, Catalog #: HCVIIA-0031), 126 μM phosphatidyl choline-phosphatidyl serine micelle and 12.5 mM $CaCl_2$ in 50mM Tris-0.15M NaCl buffer (pH 8.1). After incubation at 37°C for 9 minutes, 20 ul of 250nM human factor X was added into each incubation and incubated at room temperature for 3 minutes. Thereafter, 20 ul of 1.5 mM S-2765 chromogenic substrate (Chromogenix, Catalog #: 82 1413 39) was added. Changes of OD405nm/ 650nm were measured in kinetic mode using a SpectraMax M5 microplate reader. The standard curve was constructed by plotting Vmax against concentrations of standard using SoftMax Pro software from Molecular Device.

6. ELISA assay for measurement of plasma MECA32-Fab-TF concentration.

Plasma MECA32-Fab-TF concentration was measured using a sandwich ELISA against a purified MECA32-Fab-TF standard. Each well of ELISA plate was coated with 50 μl of 2.5 ug/ml soluble murine PLVAP protein in PBS buffer overnight at 4°C. After three washings with 250 ul washing buffer (PBS-0.2% Tween 20) and blocking with 150 μl 2% BSA in washing buffer, each well was incubated with 50 ul of MECA32-Fab-TF standard (0.01 ug/ml to 0.5ug/ml) or diluted plasma samples. After incubation for 45 minutes at room temperature, each well was washed three times and incubated with 50 μl biotinylated rabbit anti-rat IgG(H+L) antibody for 45 minutes at room temperature. After three washings and incubation with 50 ul of diluted streptavidin-alkaline phosphatase conjugate, each well was developed with 100 ul substrate for 20 minutes. OD at 405nm was measured for each well. The measured ODs for standards of different dilutions were plotted for the assay.

7. Measurement of binding affinity for MECA32-Fab-TF

The assay used to determine binding affinity between anti-PLVAP MECA32-Fab-TF and mouse PLVAP was based on the use a chromogenic TF activity assay to quantify the amounts of MECA32-Fab-TF bound to murine PLVAP in assay wells of an ELISA plate and Scatchard analysis. First, each well of an ELISA plate was coated with 2.5μg/ml water soluble recombinant mouse PLVAP over night. After washings and blocking as described for the ELISA to study MECA32-Fab-TF binding to PLVAP (Method 1), wells coated with mouse PLVAP protein were incubated with 50 μl of increasing concentrations of MECA32-Fab-TF at 0.3125, 0.625, 1.25. 2.5, 5 and 10 μg/ml in duplicates. After incubation for 3 hours at room temperature to ensure reaching a steady

state, wells were washed and assayed for amounts of TF activity bound in wells using a chromogenic TF activity assay as described earlier (Method 5). The total concentration of MECA32-Fab-TF added in each well was known and concentration of bound MECA32-Fab-TF in each well could be calculated from the TF activity assay. The TF activity of bound MECA32-Fab-TF in each well was converted to concentration based on TF specific activity of MECA32-Fab-TF and original incubation volume. The concentrations of the free and the bound MECA32-Fab-TF in each well then were used to determine steady state binding affinity (Kd) by Scatchard plot (Ann NY Acad Sci 51:660, 1949).
